# Carers’ recognition of concurrent visual impairment in dementia

**DOI:** 10.1177/13872877251372971

**Published:** 2025-09-03

**Authors:** Samantha L Tyler, Kevin B Paterson, Claire V Hutchinson

**Affiliations:** 1 School of Psychology and Vision Sciences, College of Life Sciences, University of Leicester, Leicester, UK; 2 School of Psychology, College of Life and Environmental Sciences, University of Birmingham, Birmingham, UK; 3 School of Education and Social Sciences, University of the West of Scotland, Paisley, Scotland, UK

**Keywords:** Alzheimer's disease, carers, dementia, national eye institute visual function questionnaire (NEI VFQ-25), visual activities scale (VAQ), visual impairment, vision-related quality of life

## Abstract

**Background:**

40–60% of people with dementia live with visual impairment. Those with mild-moderate dementia reliably report reduced vision-related quality of life across several visual domains with associated effects on vision-related social functioning. Despite the marked impact on concurrent dementia and visual impairment, no studies have investigated whether the impact of visual impairment on the lived experiences of people with dementia is recognised by their carers.

**Objective:**

The present study investigated whether: (1) problems related to vision and vision-related quality of life reported by people with dementia are recognised and acknowledged by their carers and (2) whether currently validated vision-related quality of life measures for mild-moderate dementia are appropriate as proxy measures of vision-related quality of life in this group.

**Methods:**

The 25-item National Eye Institute Visual Function Questionnaire (NEI VFQ-25) and the Visual Activities Questionnaire (VAQ) were completed by 48 participants with mild-moderate dementia and 48 carers. Participants with dementia completed the questionnaires based on their own experiences and carers completed them by proxy (i.e., on behalf of the person with dementia for whom they cared).

**Results:**

There was good agreement between people with dementia and their carers on both measures overall and on the majority of subscales, with no significant differences between carer and dementia reports.

**Conclusions:**

This provides further support that people with mild-moderate dementia can accurately report their visual experiences, and that carers could provide a valid proxy of the existence and impact of visual impairment on people living with dementia.

## Introduction

Age represents one of the greatest risk factors for both visual impairment (sight loss that is unresolvable with spectacle correction) and dementia. Although approximations vary between world regions, socio-economic status and gender, global estimates suggest that in 2015, around 83.24 million people over the age of 70 years were likely to be living with moderate to severe visual impairment.^1^ Equivalent figures for dementia suggest that in 2019/2020 at least 55 million older adults were likely to be living with a dementia diagnosis.^2,3^ Over the next quarter of a century, population growth and aging are likely to significantly drive the global incidence of both visual impairment and dementia further upwards. Taken together, these figures mean that many older people living with dementia will also be living with visual impairment. Recent estimates suggests that as many as between 30 to 50% of people with dementia are also visually impaired.^4^ Moreover, older adults with sight loss are more likely to have dementia compared to people with no problems with their vision.^5^

The effects of having both sight loss and dementia concurrently are more severe than those resulting from either dementia or sight loss alone^4^ and permeate many aspects of daily living.^6^ Visual impairment is associated with more cognitive decline, cognitive impairment or dementia among older adults^7^ and changes in visual function may even represent a potential early biomarker for Alzheimer's disease.^8^ In everyday life, visual impairment is likely to exacerbate existing challenges for people with dementia, an example of which includes the ability to judge social cues from facial expressions, or identify landmarks or read street signs which may help orient them and help their ability to navigate the world around them. In a wider context, visual impairment also makes it more likely that older adults, including people with dementia, will experience depression, anxiety, loneliness, reduced quality of life, reduced participation, and increased risk of falls.^9–11^

In spite of growing experimental evidence for increased rates of visual impairment in dementia, there has been relatively little research to determine its impact on the lived experiences of people with dementia. The few studies that have sought to address this issue have found that, even in the early stages of dementia, visual difficulties are commonly reported by patients which significantly impact everyday function.^5,12^ In a recent study, for example, we^12^ compared vision-related quality of life in people with mild-moderate dementia with healthy, dementia-free older adults and validated two commonly used vision-related quality of life measures: the 25-item National Eye Institute Visual Function Questionnaire (NEI VFQ-25)^13^ and a Visual Activities Questionnaire (VAQ)^14^ for use in people with mild-moderate dementia. Common themes of visual impairment and reduced vision-related quality of life included impairment when completing near and distance visual activities, impaired color and depth perception, poor peripheral vision, visual search, and visual processing speed. Reduced vision-specific social functioning was also reported.

It is promising that people in mild-moderate disease stages are still able to report that they experience visual difficulties that impact their everyday lives. However, it is worth noting that there may be groups of people with dementia for whom it is more challenging to articulate their visual impairment and/or its impact on their lives. In such cases, a reliable proxy account of visual impairment is necessary. Despite this, research has not addressed whether proxy reports on vision-related quality of life measures are reliable in a dementia population, and so there is little to no knowledge about the visual experiences of people with dementia for whom proxy report is necessary. To address this issue, here, we compare self-reported vision-related quality of life in people with mild-moderate dementia with their carers’ perceptions of those effects by proxy.

## Methods

### Participants

Participants with dementia were community-dwelling, all of whom had received a formal medical diagnosis of mild-moderate dementia, confirmed by their carer. Participants with dementia were assessed for their mental capacity to consent prior to recruitment using the Mental Capacity ACT 2005 two-stage test of capacity to ensure all participants with dementia could understand and respond to questions. Carers were only included if they knew the person with dementia well (i.e., spouse, family member, close friend, or long-term private carer) and were excluded if they had a diagnosis of dementia themselves.

One hundred and eighty-six participants were recruited to the study. Ninety-three had a diagnosis of mild-moderate dementia and 93 were the carers of the participants with dementia. Of these 186 participants, 162 (81 with dementia and their carers) were recruited from dementia support groups in Leicestershire, Rutland and Burton-on-Trent, England, UK and 24 (12 with dementia and their carers) were recruited via the Join Dementia Research online recruitment tool (https://www.joindementiaresearch.nihr.ac.uk/).

Participant response rate for the return of questionnaires was 59.14%, with 110 questionnaires returned. Only participant pairs with both a completed dementia and carer's perspective questionnaire were included in analyses. One additional participant with dementia and their carer were excluded because the participant with dementia responded ‘n/a’ to the dementia question. After exclusions, there were 96 participants in the study, 48 with dementia (mean age: 75.98 years, SD = 10.98; gender: 28 males, 20 females) and their carers. The average time since dementia diagnosis was 3.60 years (SD = 3.31). Socioeconomic status was comparable between groups. All methods adhered to the tenets of the Declaration of Helsinki and ethical approval was granted by the University of Leicester Research Ethics Committee.

### Materials

Vision-related quality of life was assessed using two measures: the 25-item National Eye Institute Visual Function Questionnaire (NEI VFQ-25)^13^ and the Visual Activities Questionnaire (VAQ).^14^ The NEI VFQ-25 consists of 25 questions organized into 12 subscales (general health, general vision, ocular pain, near activities, distance activities, vision-specific social functioning, vision-specific mental health, vision-specific role difficulties, vision-specific dependency, driving, color vision, peripheral vision). Raw scores were converted to scores ranging from 0 to 100, with lower scores indicating lower vision-related quality of life. A composite score (average of all scores except general health) was also calculated. The VAQ consists of 33 questions organized into 8 subscales (color discrimination, glare disability, light/dark adaption, acuity/spatial vision, depth perception, peripheral vision, visual search, visual processing speed). Higher scores indicate lower vision-related quality of life. A composite score (average of all scores) was also calculated. These measures have been validated for use in older adult samples^14–17^ and we have recently validated them for use in dementia.^12^

Questionnaires were administered in person (support groups) or distributed and returned by post (via Join Dementia Research and support groups if participants could not complete questionnaires during the support group session). Carers and people with dementia completed the questionnaires separately. Carers were instructed to consider whether they believed the person they cared for to have any of the problems listed in the questionnaire items and if so, what they considered the severity/frequency of that problem to be. People with dementia were asked to self-report their own vision-related difficulties via interview by a member of the research team. In some cases where questionnaires were distributed and returned by post, carers administered the questionnaire and recorded the responses of the person with dementia. When the questionnaire was administered by carers, carers were instructed to ask the exact questions to the person with dementia, providing prompts or examples where necessary, and to write down their exact responses.

### Data analyses

All analyses were performed using SPSS statistical software for Windows version 28 (IBM Corporation, Armonk, NY, USA). Analyses were conducted separately for each questionnaire. All p-values were two-tailed. Shapiro-Wilks tests of normality showed that data were not normally distributed. Therefore, questionnaire scores for the dementia and carer groups were compared using the non-parametric Wilcoxon Rank Signed statistical tests for matched-pairs and Spearman's Rho statistics. Where missing data were identified, the participant pair (dementia & carer) were removed from the analysis for a given subscale/item. Alpha levels required for significance were corrected to reflect multiple comparisons (a significance threshold of *p* < 0.01). Before conducting analysis on the NEI VFQ-25 data, the driving subscale was removed as we previously found this subscale to be an invalid measure in a dementia population, given that many participants with dementia have already given up driving.^12^ Given the novelty of this investigation, it was difficult to conduct a meaningful power calculation to assess sample size a priori. A post-hoc power analysis suggests that at a power at 0.8 and alpha of 0.01, our sample size is sensitive to effect sizes above 0.34 and so is sufficiently powered for detecting correlations of medium and large effect sizes. The smallest significant effect size detected was *r* = 0.409, indicating that our analyses were sufficiently powered to detect correlations at the observed effect sizes.

## Results

**Figure 1.**
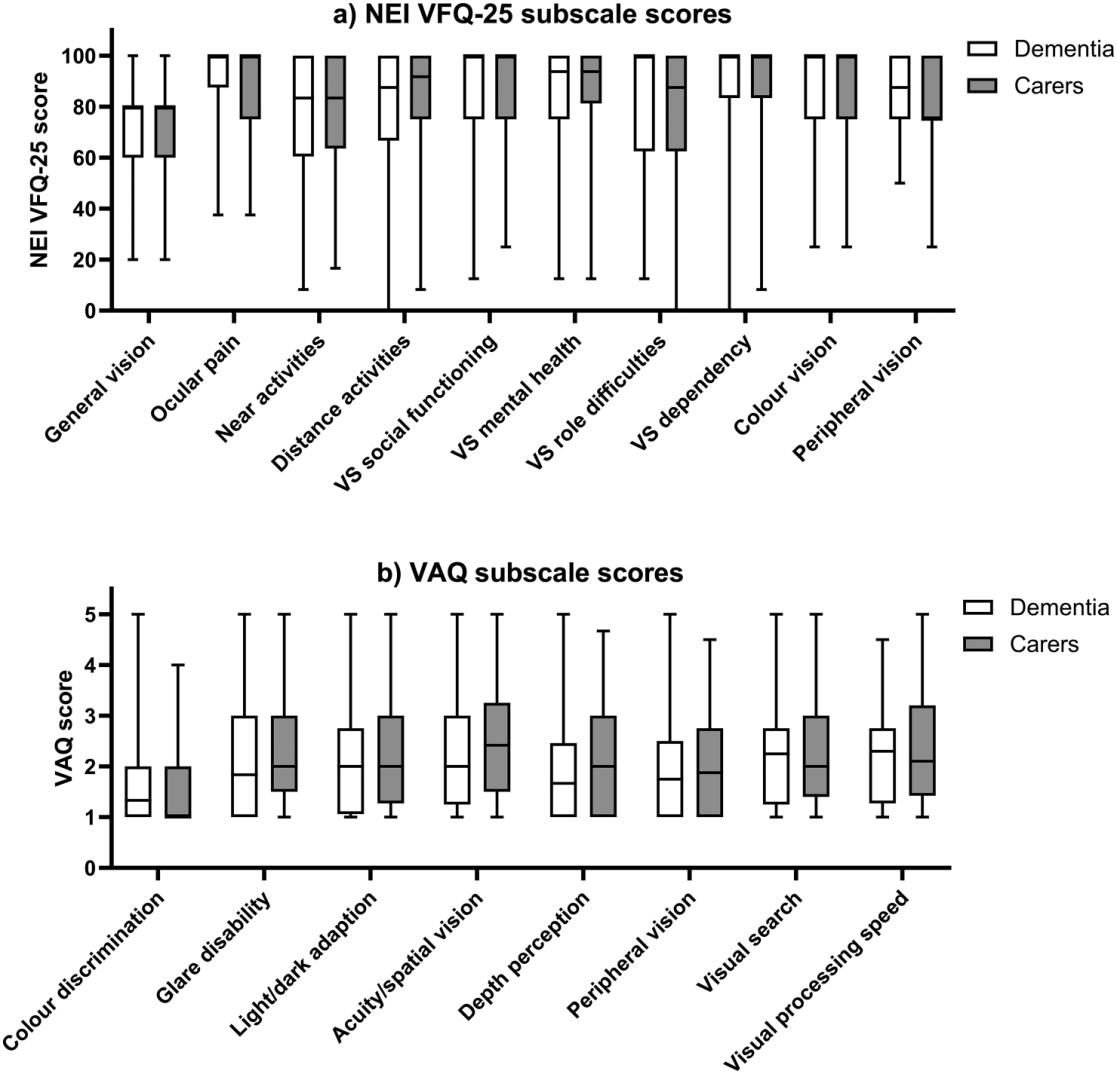
Median, interquartile range, upper and lower scores for responses of people with dementia (open bars) and their carers (closed bars) on (a) NEI VFQ-25 and (b) VAQ subscales.

Figure 1 shows scores (median, interquartile range, upper and lower scores) for (a) NEI VFQ-25 and (b) VAQ subscales. Self-reports of visual impairment varied amongst people with dementia. Importantly, for each matched pair (person with dementia and their carer), carers’ perceptions of that visual impairment were in keeping with those reported by the person with dementia. This pattern of results was confirmed using Wilcoxon Signed-Rank Tests which showed that there were no significant differences between self-reported vision-related quality of life in people with dementia and their carers’ perception of their vision-related quality of life for any subscale (Table 1).

**Table 1.** Results of Wilcoxon signed-rank tests for NEI VFQ-25 and VAQ subscales.

	No. pairs	*z* score	*p*
***NEI VFQ-25 subscales***			
General Vision	43	0.287	0.774
Ocular pain	47	−1.841	0.066
Near activities	44	−0.433	0.665
Distance activities	47	0.732	0.464
Vision-specific social functioning	45	−0.05	0.96
Vision-specific mental health	47	1.566	0.117
Vision-specific role difficulties	46	−1.153	0.249
Vision-specific dependency	46	0.489	0.625
Color vision	40	−0.22	0.826
Peripheral vision	44	−0.167	0.867
***VAQ subscales***			
Color Discrimination	47	−0.701	0.483
Glare Disability	46	1.745	0.081
Light/Dark Adaption	48	0.276	0.782
Acuity/Spatial Vision	47	0.443	0.658
Depth Perception	48	1.509	0.131
Peripheral Vision	48	−0.317	0.751
Visual Search	47	0.465	0.642
Visual Processing Speed	48	1.208	0.227

**Figure 2.**
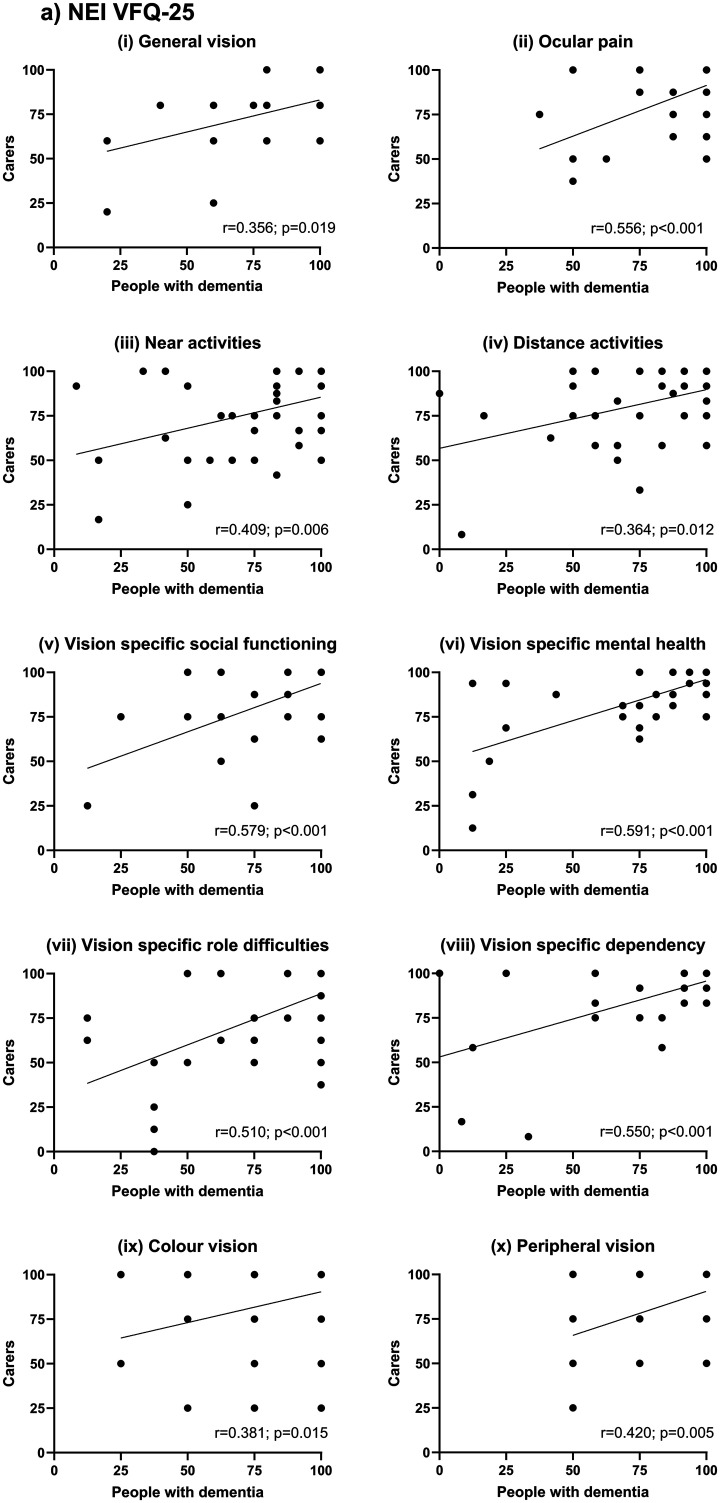

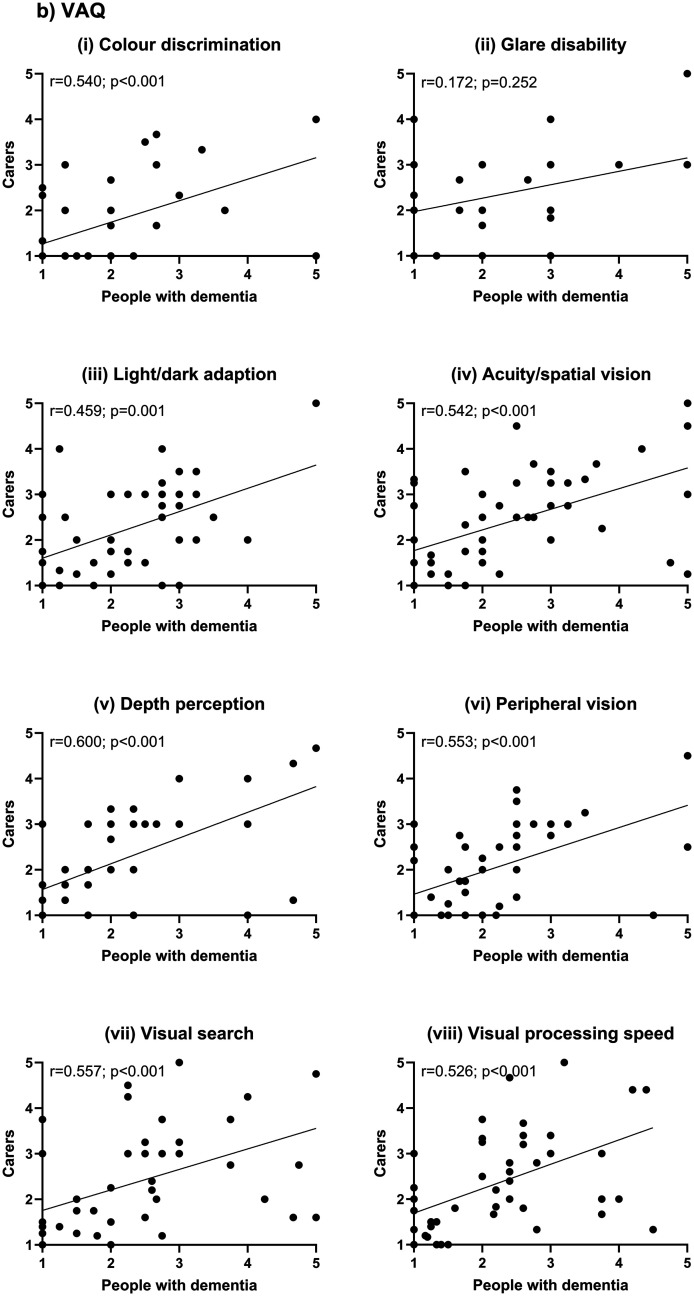
(a) NEI VFQ-25 subscale scores for self-reports of vision-related quality of life by people with dementia versus their carers’ perspective on the vision-related quality of life experienced by their loved one. Spearman's Rho statistics (*r* & *p*) for each subscale are given on the corresponding plots. Note that General Vision, Distance Activities and Color Vision were not significantly correlated according to our corrected threshold for significance (in all cases *p* > 0.01). (b) VAQ subscale scores for self-reports of vision-related quality of life by people with dementia versus their carers’ perspective on the vision-related quality of life experienced by their loved one. Spearman's Rho statistics (*r* & *p*) for each subscale are given on the corresponding plots. Note that Glare Disability was not significantly correlated according to our corrected threshold for significance (*p* > 0.01).

Figure 2 shows subscale scores for self-reports of vision-related quality of life by people with dementia versus their carers’ perspective on that vision-related quality of life on (a) the NEI VFQ-25 and (b) the VAQ. Spearman's Rho analysis (Table 2) confirmed that patient and carer perspectives were significantly associated for the averaged composite scores (NEI VFQ-25: *r*(45) = 0.622, *p* < 0.001; VAQ: *r*(46) = 0.557, *p* < 0.001) and on the majority of subscales, with the exception of General Vision, Distance Activities and Color Vision for the NEI VFQ-25 and Glare Disability for the VAQ.

**Table 2.** Results of spearman's rho correlations between dementia and carer reports for NEI VFQ-25 and VAQ subscales.

	No. pairs	*r*	*p*
*NEI VFQ-25 subscales*			
General Vision	43	0.356	0.019
Ocular pain	47	0.556	**<0**.**001**
Near activities	44	0.409	**0**.**006**
Distance activities	47	0.364	0.012
Vision-specific social functioning	45	0.579	**<0**.**001**
Vision-specific mental health	47	0.591	**<0**.**001**
Vision-specific role difficulties	46	0.510	**<0**.**001**
Vision-specific dependency	46	0.550	**<0**.**001**
Color vision	40	0.381	0.015
Peripheral vision	44	0.420	**0**.**005**
*VAQ subscales*			
Color Discrimination	47	0.540	**<0**.**001**
Glare Disability	46	0.172	0.252
Light/Dark Adaption	48	0.459	**0**.**001**
Acuity/Spatial Vision	47	0.542	**<0**.**001**
Depth Perception	48	0.600	**<0**.**001**
Peripheral Vision	48	0.553	**<0**.**001**
Visual Search	47	0.557	**<0**.**001**
Visual Processing Speed	48	0.526	**<0**.**001**

## Discussion

Here, we have shown good agreement between self-reported visual impairment and vision-related quality of life in people with mild-moderate dementia and their carers’ perceptions of that visual impairment. These findings suggest that, in cases where someone with dementia lives with concurrent visual impairment, this is likely to be recognized by their carer. These findings are important for several reasons.

Self-report measures rely on the participant's insight and understanding of their own “normal”. They require people to recall times when they have done a particular action and how they felt at the time to be able to accurately answer the question. As people with dementia have symptoms associated with memory difficulties and confusion,^18^ responses may not necessarily be an accurate reflection of their everyday visual experiences. Whether people with dementia are accurate in their self-reports on quality-of-life measures have been widely debated in the area because of these reasons.^19^ Despite these concerns, our findings of significant associations between the pattern of dementia-related visual impairment reported by people with dementia and their carers suggests that people with mild-moderate dementia can accurately report their visual experiences. These are in keeping with previous findings that people with mild-moderate cognitive impairment can reliably report information regarding their health conditions when questionnaire items and response options are simple and administered by interview.^20–22^ Additional support for this can also be seen specifically regarding quality-of-life self-reports. Several studies indicate that patients with dementia in the mild to moderate stages of the disease can rate their quality-of-life with good reliability and validity^19–21^ and self-reports on quality-of-life measures are often similar to reports by proxy particularly for items relating to health and self.^19,21^

It is generally considered best practice to measure quality-of-life directly from the individual with dementia,^20,23^ with report by proxy generally reserved for patients with severe dementia in the later stages of the disease where it is often necessary to use proxy reports.^24–26^ However, there are certain instances where report by proxy may be useful in a dementia population, particularly if communication breakdown or other barrier means that a person with dementia is no longer able to communicate their answers effectively. We provide evidence to show that the NEI VFQ-25 and VAQ can be measured via proxy in a mild-moderate dementia population. For the overwhelming majority of subscales, carer perceptions were strongly associated with the dementia participants’ own perceptions of their vision-related quality of life on both the NEI VFQ-25 and VAQ, suggesting that measures by proxy are valid when looking at overall vision-related quality of life in a mild-moderate dementia population. This is consistent with previous findings of good validity and reliability of proxy reports in a dementia population^27^ on quality-of-life measures with observable domains.

It is worth noting that the findings of this study are based on the participants that returned completed questionnaires. While our response rate of 59.14% is typical of paper surveys completed by post,^28^ there is a possibility that the 76 participants that did not return their questionnaires may have experienced different vision-related quality of life or found the questionnaires challenging to complete compared to those that had completed and returned their questionnaires. It is important, therefore, that future research into this area ensures that these reasons are not systematic in nature or due to difficulties completing the questionnaires because of their dementia.

Recognition of the co-occurrence of visual impairment and dementia is critical for improving the everyday lives of people with dementia. Visual impairment is likely to impact performance on several cognitive tests used to diagnose and monitor the progression of dementia. In isolation, both dementia and visual impairment are associated with confusion, loneliness and isolation and an increased risk of disability in older adults.^29–31^ Their co-occurrence is likely to increase this risk with associated psychological impact. It is promising therefore that the carers in our study recognized the patterns of overall visual impairment experienced by their loved ones. The findings presented here also provide additional evidence for the suitability the NEI VFQ-25 and VAQ for assessing vision-related quality of life in this group. However, wider recognition amongst healthcare providers of the prevalence of visual impairment in dementia along with strategies for addressing this important quality of life issue will be critical for helping people with dementia to live well for longer.
